# Nanoscale Characterization of Fungal-Induced CaCO_3_ Precipitation: Implications for Self-Healing Concrete

**DOI:** 10.1021/acsami.5c07137

**Published:** 2025-06-20

**Authors:** J. R. Marius Tuyishime, Edith C. Hammer, Martí Pla-Ferriol, Karina Thånell, Carl Alwmark, Sophie van Velzen, Dimitrios Floudas, Rasa Platakyte, Martin Obst, Hanbang Zou

**Affiliations:** † 226073 MAX IV Laboratory, 224 84 Lund, Sweden; ‡ 5193 Lund University, 223 62 Lund, Sweden; § BayCEER, 26523 University of Bayreuth, 95448 Bayreuth, Germany

**Keywords:** STXM, polymorph, calcium speciation, calcite, carbon speciation, bioconcrete

## Abstract

Cracks in concrete compromise structural integrity by exposing steel reinforcement to corrosion agents, shortening its service life. Fungal-induced calcium carbonate (CaCO_3_) precipitation via urea hydrolysis offers a fast and robust self-healing mechanism to seal the cracks, extending the lifespan while reducing the carbon (C) footprint of concrete infrastructure. However, current studies rely on bulk-scale analytical methods, which lack the spatial resolution and chemical sensitivity to distinguish and map CaCO_3_ polymorphs at the nanoscale. This study combined scanning electron microscopy (SEM) and synchrotron-based scanning transmission X-ray microscopy (STXM) with near-edge X-ray absorption fine structure (NEXAFS) spectroscopy to characterize fungal CaCO_3_ polymorphs at the nanoscale. CaCO_3_ biominerals precipitated by three urease-positive fungi were sectioned into 75–200 nm thin layers. STXM data were collected from at least two spots per section, focusing on Ca (L-edge) and C (K-edge) chemical speciation and elemental quantitative mapping. Calcite, the thermodynamically most stable polymorph, was identified as the predominant mineral phase precipitated by all fungi species, while aragonite and non-CO_3_–Ca species (CaCl_2_ or Ca adsorbed onto extracellular polymeric substances (EPS)) occurred as minor components. In fungal species 2, we observed nanoscale heterogeneity in Ca phases across five analyzed spots, three dominated by calcite with minor contributions of other Ca species, while the others showed mixed CaCO_3_/non-CO_3_ phases, as confirmed by NEXAFS spectra. These findings suggest that biomineralization in the fungal micro and nanoenvironment is influenced by localized physicochemical and metabolic conditions that shape mineral phases. C NEXAFS spectra further supported the Ca data, showing C-specific spectral features in the calcite-rich regions across all samples. This underscores STXM’s capability to resolve complexities and mechanisms of fungal CaCO_3_ formation (e.g., mineral phase composition, fungal organic-mineral interactions, and spatial heterogeneity). Overall, this study provides critical nanoscale insights into fungal CaCO_3_ precipitation, thus providing valuable guidance in optimizing fungal systems in self-healing concrete applications.

## Graphical abstract

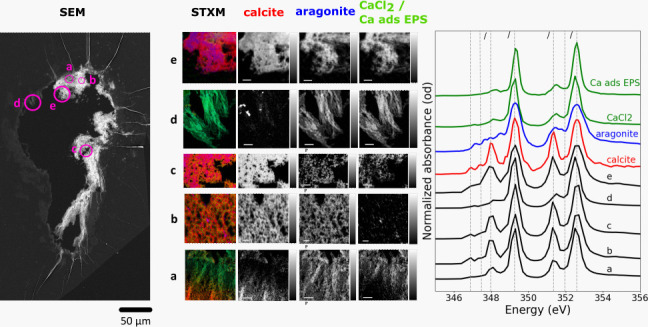

## Introduction

Concrete is the most widely used human-made material worldwide. Despite its ubiquitous presence, its environmental credentials have come under increased scrutiny in the last couple of decades, as its production requires large amounts of energy and contributes about 8% of global carbon dioxide (CO_2_) emissions.^1^ The lifetime of a concrete structure is often determined by when the steel reinforcement will start to corrode, either because of carbonation from reaction with CO_2_ or the ingress of chlorides from deicing agents.^2^ As the concrete matrix is a brittle material prone to cracking, healing these cracks is essential to preventing reinforcement corrosion. Bioinspired self-healing approaches hold a great promise,^3,4^ but the underlying biomineralization mechanisms, especially at subcellular level, are still not well understood.

Using microbes to heal concrete cracks is an innovative, sustainable, and safe idea that has come up in recent years.^5,6^ One of the common microbial self-healing solutions consists of spheres that contain the microbe and nutrients in dry form embedded into the concrete. Upon rehydration at cracking in the concrete, the microbes become activated and seal the cracks through their growth and microbially induced precipitation of CaCO_3_ (MICCP) that is intrinsic to concrete compositions.^7,8^ While photoautotrophic microorganisms (e.g., cyanobacteria, micro algae) require light as an energy source, promoting CaCO_3_ precipitation, and have been used successfully to restore surfaces (e.g., limestone monuments),^9^ they are not suitable inside concrete structures. CaCO_3_-forming chemotrophic bacteria have been successfully demonstrated to heal the smallest (up to 1 mm) cracks in concrete,^10^ but they are locally refined to their microlocation as they have very limited dispersal capability in the concrete matrix. Fungi, by contrast, offer distinct advantages: they form resilient, mesh-like hyphal networks that enhance CaCO_3_ precipitation and can thrive in extreme conditions, including high alkalinity and nutrient scarcity, which are typical of concrete and other similar environments such as mortar.^11,12^

Numerous studies have employed laboratory-scale approaches with urea-based media to investigate the capacity of microorganisms, including fungi, to precipitate CaCO_3_ minerals and their relevance for bioconcrete. Bulk X-ray diffraction (XRD) technique has proven effective in identifying the crystal structures, crystallinity, and mineralogical phases of these precipitates.^13,14^ However, this technique offers bulk-scale structural information, requiring additional methods to gain a more detailed mechanistic understanding of biomineralization. Scanning electron microscopy (SEM) is commonly used to visualize surface morphology and structural characteristics. Coupling SEM with energy-dispersive X-ray (EDX) spectroscopy enhances its ability to reveal elemental abundance and distribution within biomineralized structures. In addition, Fourier-transform infrared (FTIR) spectroscopy has been useful in identifying biochemical pathways linking crystal growth and microbial metabolism, including studies linked to concrete self-healing.^15,16^ Despite their wide application, these methods face inherent limitations in terms of spatial resolution or chemical sensitivity, which restrict their ability to resolve nanoscale features and distinguish among specific compounds.

Synchrotron-based scanning transmission X-ray microscopy (STXM) combines high spatial resolution imaging (<30 nm) with elemental speciation analysis by near-edge-absorption fine structure (NEXAFS) spectroscopy, which makes it an ideal tool to study samples that are inhomogeneous at nanometer or micron scales. It is a transmission-mode technique that scans samples across specific X-ray absorption edges to generate a sequence of images, which serve as a basis for generating NEXAFS spectra, thus linking structural imaging with chemical characterization. It achieves this by exciting core electrons (e.g., K, L shells) of a target element to higher unoccupied molecular orbitals or continuum states.^17^ For instance, Ca NEXAFS displays two main spin–orbit-related absorption peaks, L_3_ and L_2_, along with preceding peaks that arise due to variations in the local symmetry of atomic arrangements around Ca atoms.^18^ The number, intensity, and position of these two crystal-field-related peaks preceding the main resonance edges are very crucial for distinguishing between Ca species of different CaCO_3_ polymorphs or other Ca compounds.^19^ Similarly, STXM is an effective technique to study C speciation, (e.g., ref 20). The presence of the CO_3_^2–^ functional group is indicated by a sharp resonance peak at the C K-edge, typically at ≈290.3 eV, corresponding to C 1s → π*_C=O_ electronic transitions, along with a small postedge peak at ≈295 eV and a broad postedge peak at ≈301.3 eV, both associated with 1s core → σ* transitions.^21–23^ However, differences in CaCO_3_ are primarily observed through subtle variations in the post- and pre-edge spectral features, especially when other organic functional groups with overlapping or distinct peak positions,^24,25^ are associated with polymorphs. In the field of CaCO_3_ biomineralization, the application of STXM has provided valuable insights. For instance, STXM was utilized to study the planktonic cyanobacteria of the strain *Synechococcus leopoliensis* PCC 7942 and identified two CaCO_3_ precipitation pathways: (i) formation of amorphous CaCO_3_ biominerals (identified as aragonite) within the EPS regions under CaCO_3_-supersaturated conditions, serving as a protective mechanism against (ii) uncontrolled calcite precipitation on the cell surfaces as aragonite dissolves.^26^ The application of STXM further demonstrated that calcite can form through phase transitions from transient amorphous CaCO_3_.^27^ In a different research focus, STXM was employed and spatially resolved changes in C and Ca spectral features caused by interactions between organic substances and Ca^2+^ cations from calcite dissolution.^28^ Their work highlighted the role of calcite in the chemical stabilization of organic matter. Despite these advancements, the role of fungi in biomineralization has not been studied on such a small scale, highlighting a critical gap in the literature.

As shown in Figure 1, fungi are shown to induce the precipitation of CaCO_3_ at micro spaces but in various crystal structures.^29^ In Figure 1A, CaCO_3_ coupled with fungal mycelium appears more dispersed than the denser precipitation observed in Figure 1B, where the latter appears far more effective at healing cracks. The underlying mechanisms behind these variations remain unknown. In this study, we applied STXM as a nanoscale spatial characterization technique to examine (i) the specific CaCO_3_ polymorphs formed by urease-positive filamentous fungi through Ca L-edge and C K-edge chemical speciation and quantitative mapping and (ii) the role of fungi and their organic metabolites in inducing or influencing the identified mineral phases. We aim to uncover the nanoscale mechanisms driving fungal biomineralization, providing novel insights that could guide the application of fungi in self-healing concrete technologies.

**Figure 1:**
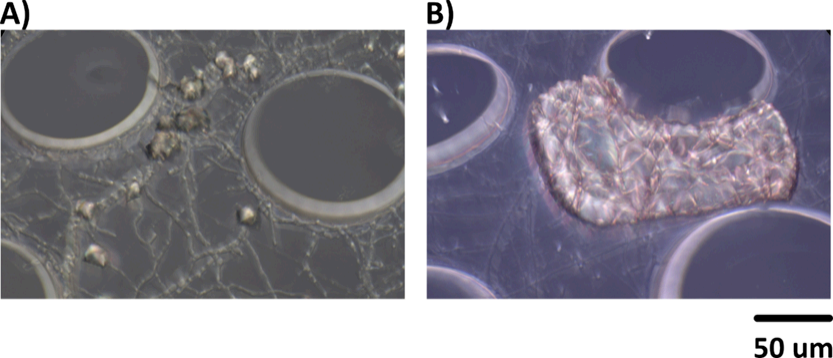
(A,B) 3D reconstructed microscopic z-stack images of calcium carbonate precipitation integrated with fungal mycelium by different fungal stains within microfluidic chips.

## Materials and Methods

### Screening and Identification of Fungal Urease Activity for CaCO_3_ Precipitation

Environmental samples were obtained from an abandoned limestone quarry in Skåne, Sweden, and brought to the Microbial Ecology Lab in the Department of Biology of Lund University. From these samples, three different urease-positive ascomycetes were isolated and identified by sequencing, hereafter referred to as fungal species 1, 2, and 3 (details in the SI). The three fungal candidates were validated and shown to be tolerant of highly alkaline conditions and compatible with cementitious materials. The selected candidates were then further studied for their CaCO_3_-precipitating activity. A small cut from an axenic culture of each fungal candidate was added to a 50 mL centrifuge tube containing a culture medium (Christensen’s Urea Broth)^30^ with Ca^2+^ ions, introduced via the addition of a 50 mM CaCl_2_·4H_2_O solution. Subsequently, all cultures were incubated at 20 °C with agitation for 14 days.

### Thin-Section Preparation from Fungal CaCO_3_ Biominerals

Following incubation, samples, consisting of fungi and mineral mixture, were pretreated and sectioned to produce ultrathin cuts suitable for high-resolution X-ray imaging and spectroscopy analyses in transmission mode, STXM. For samples from fungal species 1 and 2, the excess liquid medium was aseptically removed from centrifuge tubes. A portion of the remaining material (≈1 × 1 × 3 mm^3^ volume) was fixed with Karnovsky fixative (Polyscience Europe GmbH, Eppelheim, Germany) for 3 h in the fume hood to preserve some cellular structures and mineral deposits. The fixative was subsequently removed, and samples washed off with 0.1 M Sorensen phosphate buffer, followed by dehydration in acetone and embedding in low-viscosity epoxy resin (Polyscience Europe GmbH, Eppelheim, Germany). To compare the influence of two different preparation methods on thin-sectioning, the fungi-mineral complex from species 3 was oven-dried at 65 °C for 2 days, embedded in Eponate 12 epoxy resin, and cured at 60 °C for 24 h. Ultrathin sections were obtained using an ultramicrotome (Leica EM UC7) at the Soft X-ray Chemical Lab at MAX IV laboratory in Lund, Sweden. Section thicknesses achieved were 75–100 nm for fungal species 1, 100 nm for species 2, and 200 nm for species 3. Sections were mounted on silicon nitride membrane windows and 150 mesh Cu-TEM grids, in such a way that a blank area was left on the windows or grids, which helped to collect the total X-ray transmission (*I*_0_).

### Ca L-Edge and C K-Edge Speciation by STXM with NEXAFS Spectroscopy

STXM data were collected on the SoftiMAX beamline (energy range: 275–2500 eV), MAX IV Laboratory.^31^ The beamline consists of advanced X-ray beam focusing elements, including mirrors, which precisely direct and focus the incoming monochromatic beam through the exit slit, which selects the required X-ray beam shape and size before it enters the STXM chamber. Inside the chamber, a Fresnel zone plate enabled diffraction-limited focusing of the beam through the sample. The X-ray transmission photons were detected by a P47 scintillator attached to a photomultiplier tube (PMT) detector.

To obtain chemical information, ultrathin sections were raster-scanned across a nanofocused X-ray beam in the x and y directions at discrete incident energies (E) tuned around the X-ray absorption edges of Ca and C. Data were collected over the Ca L_3,2_ (2p)-edge (340–360 eV) and C K (1s)-edge (280–325 eV), with energy steps of between 0.1–0.2 eV around the absorption peaks to capture detailed information and relatively larger steps elsewhere. This process generated an image stack data set with spatial (*xy*) and E dimensions (2D, E), each image corresponding to a specific energy. This data type enables the observation of morphological and structural features as well as chemical speciation through NEXAFS. Images were recorded with a step size of 50–100 nm. For each Ca and C image stack, we acquired images at 65 and 98 photon energy points, respectively.

### STXM Data Processing and Analysis

STXM data were processed and analyzed using the Analysis of X-ray Microscopy Images and Spectra (aXis2000) software package.^32^ Initially, image stacks were inspected to identify and remove artifacts and any poor-quality images. All images were then aligned to correct for misalignment due to, for example, drift. The measured transmitted photon flux was normalized into a linear absorbance (optical density) scale using the Lambert–Beer law (eq 1):

$$\mathrm{OD}=-\ln\left(\frac{I}{I_{0}}\right)$$ 1

where *I*_0_ denotes the transmitted photon intensity through an empty area adjacent to the sample, and *I* is the detected X-ray transmission through the sample.^33^ This data transformation allows the generation of quantitative chemical species maps.

The acquisition of the (2D, E) image stack enabled the extraction of chemical information from individual images as well as NEXAFS spectra from specific pixels or regions, including thickness-based data. For Ca, masks were generated from an average image stack across the Ca L-edge by selecting pixels with OD values below 0.5 but above the noise threshold (set to 0.05). This selection helped minimize the influence of thicker regions prone to X-ray absorption saturation.^19^ For the oven-dried sample, which was 200 nm thick, higher OD limits were occasionally selected due to the absence of a detectable Ca signal and noticeable background noise below 0.45 OD. Similarly, for C data, spectra were derived from regions with OD values ranging from just above the noise threshold (identified individually for each dataset) to 0.5.

For quantitative chemical mapping of identified Ca and C species, sample spectra were initially fitted by using the CGO curve fit function. The function performs spectrum-by-spectrum fitting to determine the optimum combination of reference compounds that accurately represent the sample. Five Ca references were used: pure calcite, pure aragonite, pure hydroxyapatite, Ca^2+^ adsorbed on extracellular polymeric substances (Ca ads EPS), and CaCl_2_ (Figure S1). The latter two were included in the model to account for nonspecific Ca background contributions. For C, seven references were included: calcite-C, aragonite-C, xanthan gum, lipid, alginate, albumin, and epoxy resin (Figure S2). All these were collected at the Canadian Light Source (CLS) in Saskatoon, Canada, and previously normalized to an absolute linear absorbance scale (OD per nm effective thickness),^19,34–36^ except for CaCl_2_ and epoxy resin, which were obtained as internal standards from image stacks. After CGO fitting, the best three standards were selected to generate quantitative chemical species maps using linear least-squares fitting (stack fit function) applied to the image stack. To further minimize the thickness-based absorption saturation effect, three images around each main resonance peak were removed from the stack before the fitting process. This procedure was applied to each data set if the average spectrum extracted from nonsaturated regions showed OD above 1. Before performing the fitting, energy shift correction was applied to align data with the pure calcite absorption edge, typically L_3_ peak for Ca data and the C K-edge for C data. The Ca spectra were shifted by approximately 0.9 eV, and C data by 0.59 eV, toward higher energy values. The alignment ensured consistency between the STXM and reference spectral energy positions.

### SEM

Secondary electron and back scattered electron images of the samples prepared as thin sections from CaCO_3_ biominerals from fungal species 1, 2, and 3 were obtained using a Mira3 high-resolution field emission gun scanning electron microscope from Tescan, equipped with secondary and backscattered electron detectors. The investigated thin sections were coated with a 7 nm thick layer of palladium/platinum prior to analyses. Elemental analysis and elemental maps were acquired using an energy-dispersive X-ray spectrometer (EDS; X-MaxN 80 Oxford Instruments) and analyzed using the software program Aztec. The SEM operated under high vacuum, with an acceleration voltage of 15 kV, a working distance of 15 mm, and a beam current of 1–2 nA.

## Results and Discussion

This study examined the potential of urease-positive fungi to precipitate CaCO_3_ minerals, as demonstrated by STXM/NEXAFS and SEM data obtained from CaCO_3_ biominerals formed during the incubation of three selected fungal species cultured in a medium containing 50 mM CaCl_2_. SEM images revealed the morphological features of thin sections from fungal species 1 (Figure 2), species 2 (Figures 3 and S3), and species 3 (Figure S4).

**Figure 2:**
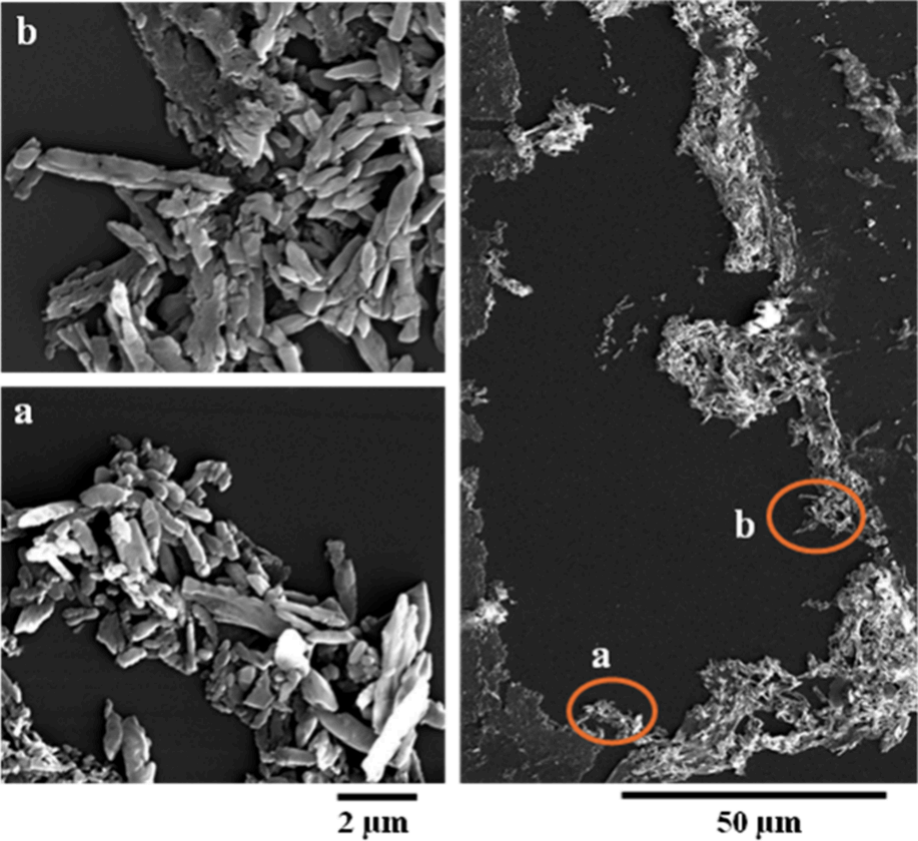
Secondary electron images showing morphological features of a thin section of CaCO_3_ biominerals produced by fungal species 1. (a) and (b) indicate areas where STXM data were collected to analyze Ca at the L-edge and C at the K-edge.

**Figure 3:**
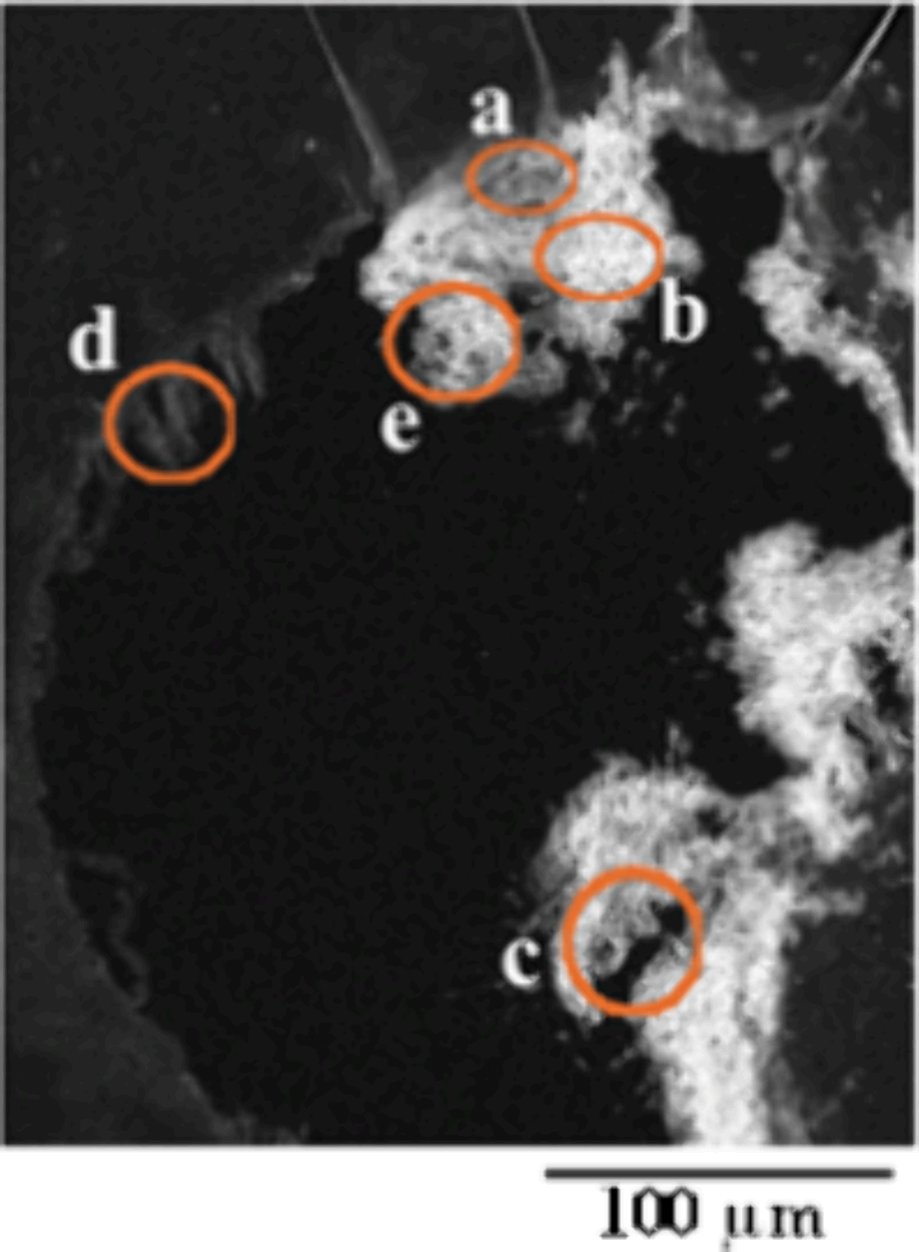
Overview of secondary electron images showing morphological features of a thin section of CaCO_3_ biominerals produced by fungal species 2. (a), (b), (c), (d), and (e) indicate areas where STXM data were collected to analyze Ca at the L-edge and C at the K-edge.

STXM/NEXAFS analyses provided critical insights into the chemical state and quantitative distribution of Ca and C species within precipitated CaCO_3_ biominerals, allowing for the differentiation of polymorphs and a deeper understanding of the mechanisms involved. Our results, which confirm calcite as the predominant CaCO_3_ polymorph precipitated via the ureolytic pathway after approximately a two-week incubation period, align with previous research on the role of fungi in biogeochemical CaCO_3_ cycles in natural environments.^37,38^ Additionally, while this work was not directly investigated in concrete and mortar, our findings contribute nanoscale insights into fungal-driven biomineralization processes relevant to such applications (e.g., ref 39). However, unlike these bulk-scale studies, our work used the nanoscale spatial resolution of STXM and NEXAFS and uncovered distinct heterogeneity in composition, with localized regions of non-CO_3_–Ca species (green), transient aragonite-like phases (blue), and the dominant calcite-like polymorphs (red), suggesting spatially variable physicochemical influences on nucleation dynamics within fungal microenvironments.

### Characterization of Ca Speciation in CaCO_3_ Biominerals

The NEXAFS analysis at the Ca L-edge enabled identification of different specific CaCO_3_ polymorphs and non-CO_3_–Ca species (Figures 4B and 5B). The Ca spectra (at the L-edge), particularly the splitting of L_3_ and L_2_ into main absorption peaks and prepeaks, served as a key distinguishing factor among Ca phases, differentiating calcite, aragonite, and CaCl_2_/Ca adsorbed onto extracellular polymeric substances (Ca ads EPS). For simplicity, we refer to the splitting of the main L_3_ peak as L_3a_ and L_3b_ components, and the main L_2_ peak as L_2a_ and L_2b_ components, where “a” denotes the prepeaks and “b” the main resonance peaks. On the other hand, Figures 4A and 5A illustrate color composite maps for quantitative chemical mapping of CaCO_3_ biominerals from fungal species 1 and 2, spectroscopically identified as calcite (red) and aragonite (blue), along with non-CO_3_–Ca compounds (green). However, given that the CGO fits yielded nearly identical results when incorporating a CaCl_2_ reference spectrum or Ca ads EPS, we collectively referred to these two non-CO_3_–Ca species as “CaCl_2_/Ca ads EPS”, as their spectral features are nearly indistinguishable (see Ca reference data in the SI).

**Figure 4:**
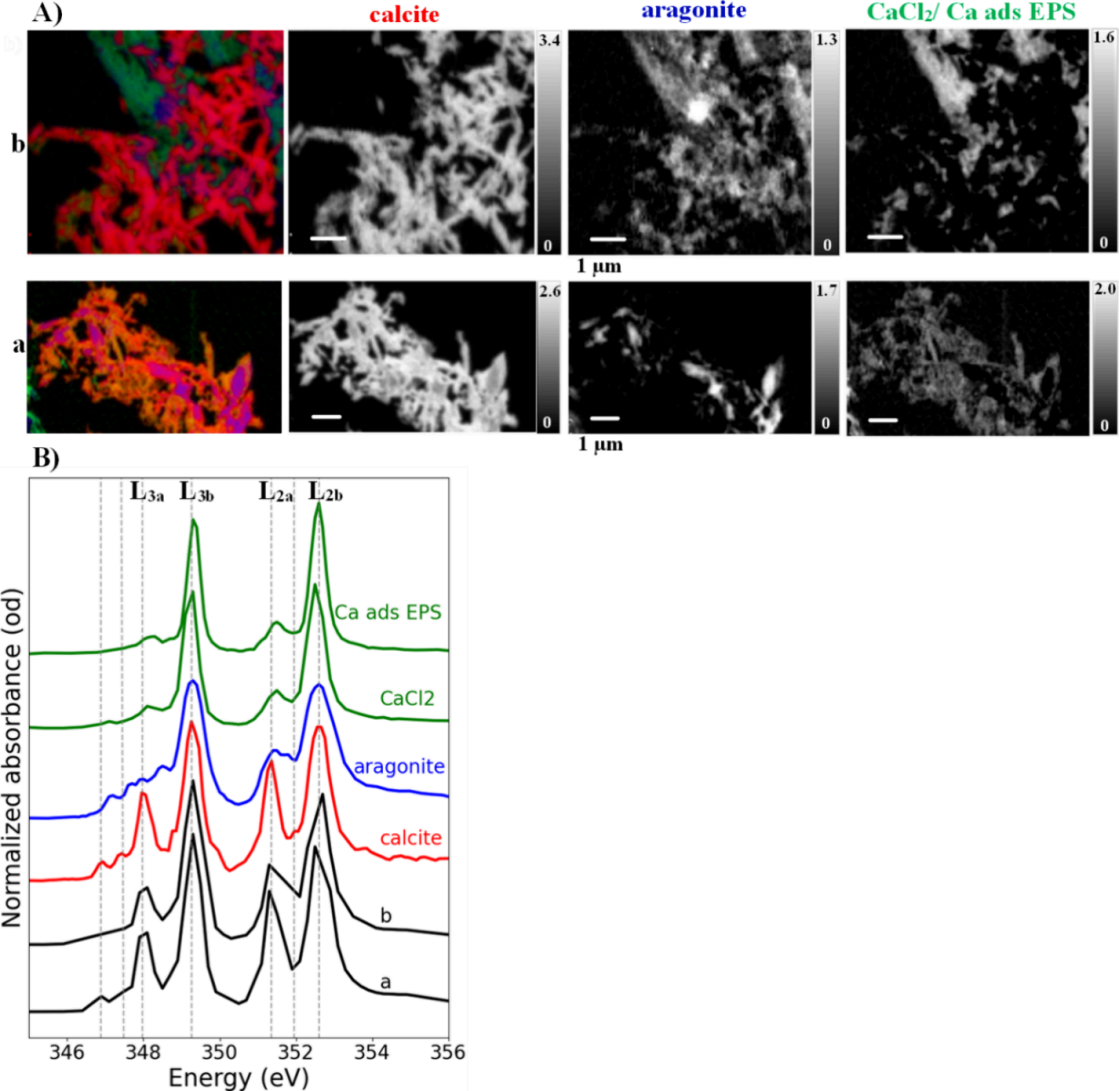
Ca L-edge STXM/NEXAFS data for CaCO_3_ biominerals produced by fungal species 1: (A) color composite maps showing Ca speciation distribution (first column) obtained by overlaying grayscale STXM quantitative maps (second, third, and fourth columns) of different Ca species in analyzed spots (a) and (b). The grayscale images, expressed in optical density, represent quantitative Ca species maps derived from fitting STXM image sequence at the Ca L-edge using Ca reference spectra. (B) Average Ca L-edge NEXAFS sample spectra (dark) extracted from regions without absorption saturation at (a) and (b) spots. The reference spectra used for stack fitting include pure Ca carbonate phases: calcite (red), aragonite (blue), along with internally derived noncarbonate Ca species, CaCl_2_, or Ca adsorbed on extracellular polymeric substances (green).

**Figure 5:**
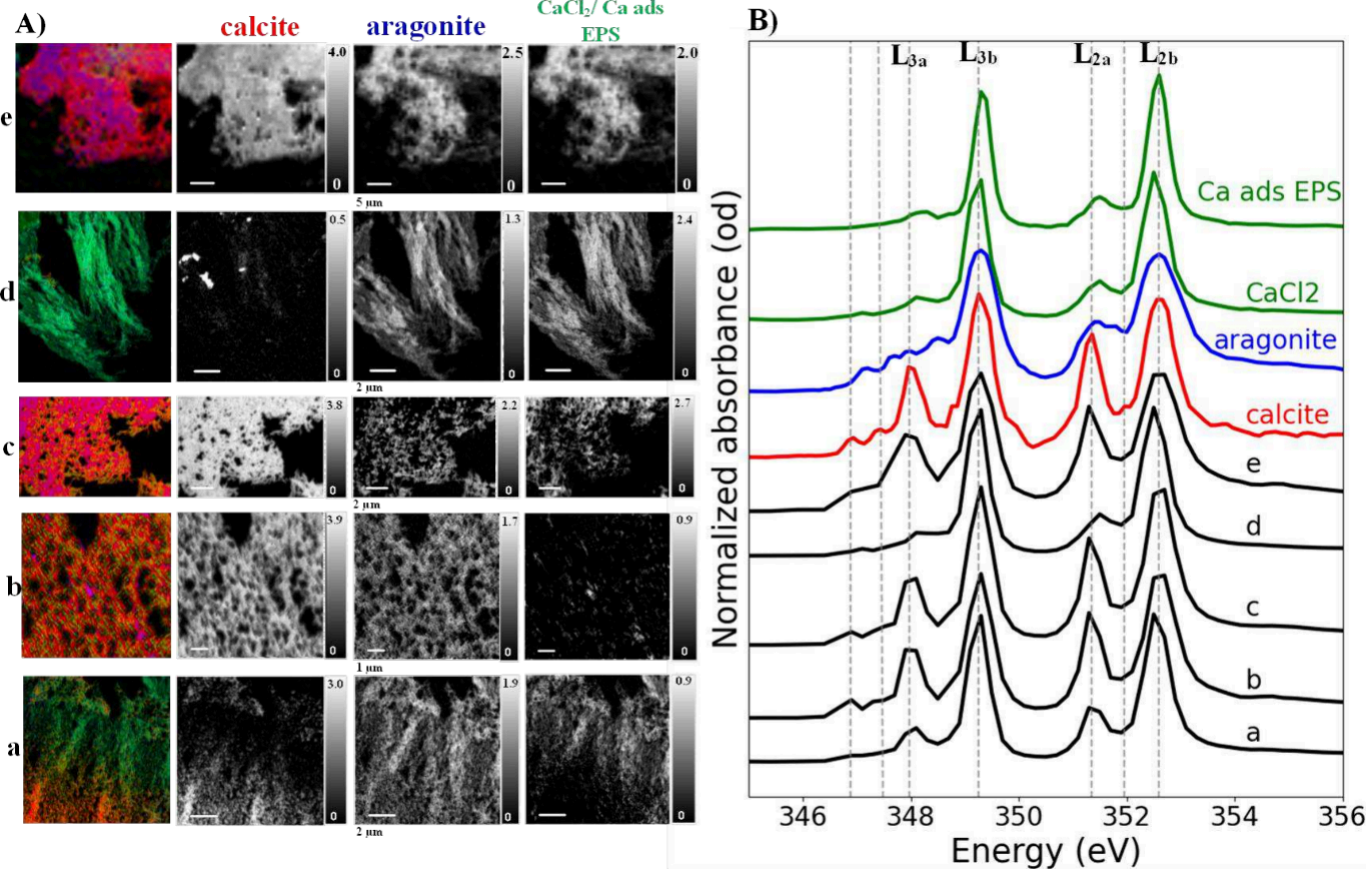
Ca L-edge STXM/NEXAFS data for CaCO_3_minerals produced by fungal species 2: (A) color composite maps of Ca speciation distribution (first column) obtained by overlaying grayscale STXM quantitative maps (second, third, and forth columns) of Ca species at analyzed spots (a), (b), (c), (d), and (e). The grayscale images, expressed in optical density, represent quantitative Ca species maps derived from fitting STXM image sequence at the Ca L-edge using Ca reference spectra. (B) Average Ca L-edge NEXAFS sample spectra (dark) extracted from regions without absorption saturation at those five spots. The reference spectra used for stack fitting include pure Ca carbonate phases: calcite (red), aragonite (blue), along with internally derived noncarbonate Ca species, CaCl_2_, or Ca adsorbed on extracellular polymeric substances (green).

All NEXAFS exhibited two main resonance peaks at the Ca L_3,2_-edge, which is a common characteristic of Ca-containing compounds. In most spots from analyzed thin sections, calcite was the predominant mineral phase, confirmed by its characteristic NEXAFS peaks: L_3b_ at 349.3 eV and L_2b_ at 352.5 eV, along with two prepeaks, L_3a_ at 348.0 eV and L_2a_ at 351.1 eV, which are relatively more intense than those observed in aragonite and other Ca species, as shown by a previous study.^19^ Minor fractions of aragonite and CaCl_2_/Ca ads EPS were also detected, but their spatial distribution varied across fungal species.

For fungal species 1, NEXAFS spectral data confirmed that calcite was the predominant polymorph, while the aragonite-like polymorph and non-CO_3_–Ca phases were found localized in specific sample thin-section areas, particularly in spot b (Figure 4). The presence of CaCl_2_/Ca ads EPS in some regions suggests early-stage Ca^2+^ accumulation in EPS regions through adsorption, which created Ca reservoirs prior to CaCO_3_ precipitation. These results reflect biomineralization pathways observed in corals, where the centers of calcification (COC) serve as nucleation zones for CaCO_3_ precipitation, followed by growth of aragonite fibers, as revealed by Benzerara and co-workers.^21^ Notably, in this study, aragonite was detected in thicker sample areas with higher OD compared to regions dominated by CaCl_2_/Ca ads EPS (see quantitative grayscale composition maps; Figure 4A). This pattern was also observed in SEM images, further supporting the localized distribution of specific mineral phases. This suggests that Ca^2+^-rich nanoregions led to localized CaCO_3_ supersaturation, potentially inducing the nucleation of transient aragonite-like CaCO_3_. This aligns with previous findings^26^ on cyanobacterial CaCO_3_ precipitation, where amorphous aragonite-like phases initially formed within EPS regions under CaCO_3_ supersaturation before dissolving and recrystallizing into calcite. Overall, our data emphasize the complex and dynamic pathways of fungal CaCO_3_ precipitation under biologically influenced conditions.

For fungal species 2, nanoscale heterogeneity in Ca phase distribution was more evident across five analyzed spots. Calcite polymorph precipitation dominated in three regions, (b), (c), and (e), whereas CaCl_2_/Ca ads EPS and aragonite were only minor fractions (Figure 5). In contrast, mixed CaCO_3_-/non-CO_3_–Ca phases were observed in spot (a), where both Ca forms precipitated homogeneously in distinct sample areas (Figure S5). On the other hand, spot (d) exhibited a predominant non-CO_3_–Ca composition, as indicated by spectra with less intense L_3a_ and L_2a_ peaks compared to pure calcite.

These observations across all five spots suggest possible variations in local metabolic activity, Ca^2+^ concentration gradients, and pH fluctuations, which likely influenced the selection of polymorph precipitation.

For the sample from fungal species 3, which was oven-dried before sectioning into a 200 nm thin section, calcite was similarly identified as the dominant form. It is worth noting that due to increased spectral noise, the absence of detectable Ca signal in low OD regions, as well as thickness-related constraints, spectra were extracted only from OD regions between 0.45 and 0.65 (Figure 6). Applying this threshold limited the Ca NEXAFS peak absorbance to a maximum of 0.75, ensuring sufficient signal while minimizing problems with absorption saturation (Figures S6 and S7).

**Figure 6:**
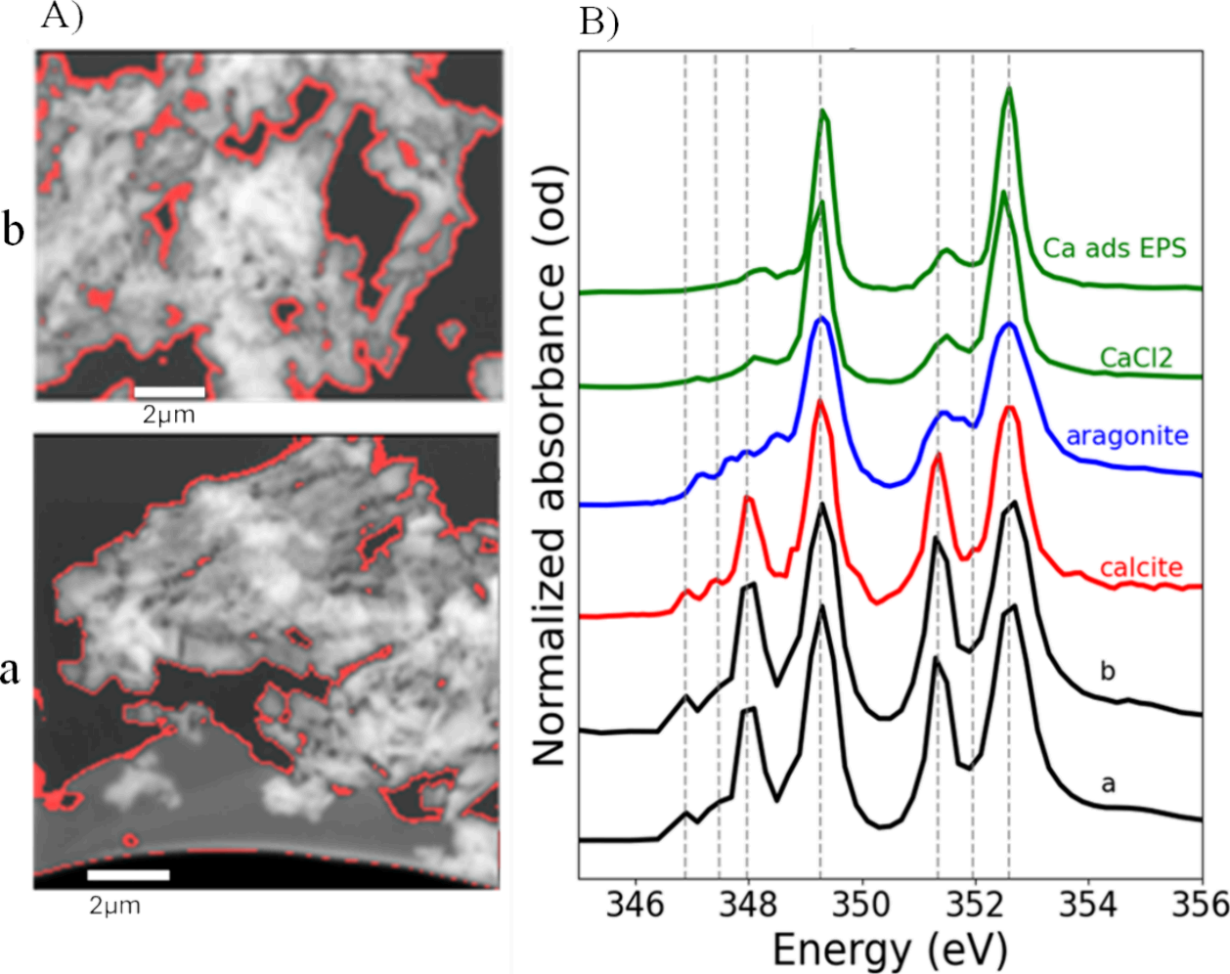
(A) Thickness-based masks (red) applied to regions with optical densities (OD) between 0.45–0.65, generated from average STXM images at Ca L-edge (340–360 eV) for spots (a) and (b) in the analyzed thin section from fungal species 3. The masks below 0.45 OD showed a very high noise level and no detectable Ca signal, while thicker regions (>0.65 OD) were excluded due to a pronounced X-ray absorption saturation. (B) Corresponding Ca L-edge NEXAFS spectra extracted from masked regions (black). The reference spectra used for stack fitting include pure Ca carbonate phases: calcite (red), aragonite (blue), along with internally derived noncarbonate Ca species, CaCl_2_, or Ca adsorbed on extracellular polymeric substances (green).

### Characterization of C Speciation in CaCO_3_ Biominerals

To complement Ca L-edge analysis in characterizing CaCO_3_ precipitates, C-edge data were collected. Initially, CGO curve fit was performed using three C references at a time for each sample spectrum. This approach helped identify representative compounds for constructing chemical C composition maps. When fitting became challenging, possibly due to noise or artifacts, spectral features were visually analyzed and compared to C references to infer the C speciation.

For fungal species 1, the C composition was primarily calcite (Figure 7A). Similarly, spectra from (b) and (c) spots and, to some extent, (a) in fungal species 2 closely matched the characteristic C spectral features of calcite. The presence of a sharp peak at ≈290.3 eV, and a smaller, more resolved peak at ≈295.2 eV, along with a broad postedge peak at ≈301.3 eV, indicates carbonate signatures consistent with calcite or aragonite. However, the presence of an additional peak at 298.5 eV in the postedge region suggests that the spectra most likely correspond to a calcite-like polymorph,^28^ as this peak is absent in the aragonite reference spectrum. In contrast, spectra from spot (d) and, to some extent, spot (a) in species 2 displayed other spectral characteristics, likely attributed to epoxy contribution (see C reference data in the SI). Data from spot (e) are not available. In fungal species 3, C NEXAFS spectra from two analyzed spots showed additional peaks around 288.2–289.5 eV (Figure S8). Along with the observed CO_3_-related spectral features, these spectral characteristics suggest a clear contribution from EPS components, such as polysaccharides, proteins, or lipids,^40^ to the chemical C composition in this sample. In summary, the C data from calcite-dominated spots in the analyzed samples align with the Ca L-edge data, further confirming the predominance of calcite in these locations.

**Figure 7:**
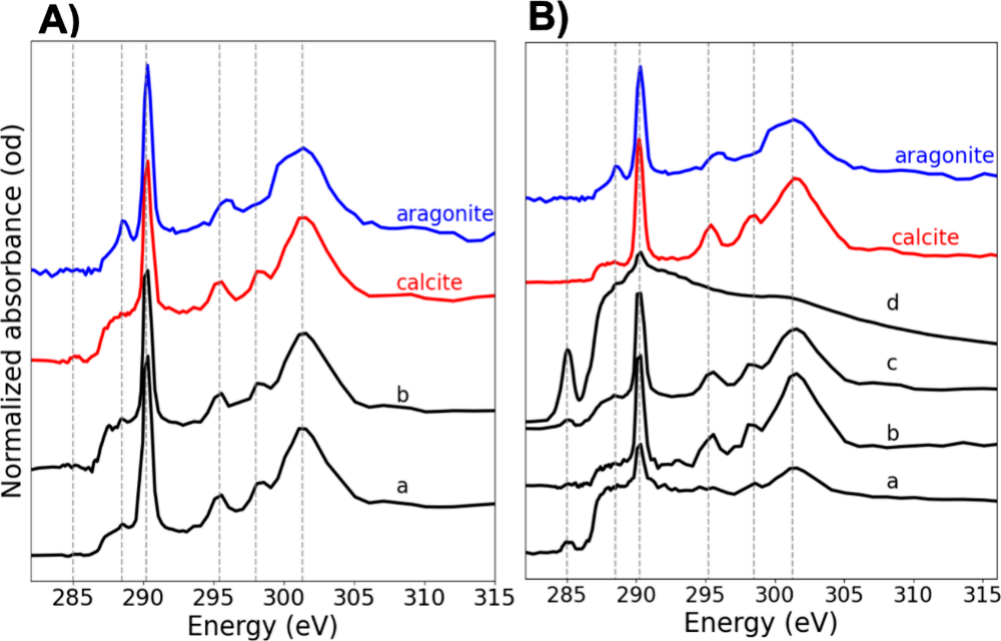
(A) C K-edge NEXAFS data for CaCO_3_ minerals produced by fungal species 1: (a) and (b) correspond to regions in Figure 2 and represent average spectra (black) extracted from nonabsorption saturation regions. (B) C K-edge NEXAFS data for CaCO_3_ minerals produced by fungal species 2: (a–d) correspond to regions in Figure 3 and represent average spectra (black) from four different analyzed spots. The sample data (black lines) are compared with the reference spectra for calcite-C (red) and aragonite-C (blue).

### Implications for Fungal Biomineralization and Self-Healing Concrete Application

The observed variations in CaCO_3_ polymorphs and non-CO_3_–Ca species highlight the complex interplay among fungal metabolism, physicochemical conditions, and organic matrices in biomineralization. In fungal species 1, aragonite-like phases were primarily localized in regions that extend from Ca ads EPS, likely within EPS-rich areas. The locally elevated Ca^2+^ concentrations in these regions facilitated transient aragonite nucleation, which led to increased mineral density. A similar biomineralization pathway was observed in cyanobacteria, where amorphous aragonite precursors initially form within EPS before transforming into stable calcite as the supersaturation is no longer sustained.^26^ The results also reflect a progression from mineral nucleation driven by supersaturation to calcite crystal growth under undersaturation conditions with respect to CaCO_3_.^41^ For fungal species 2, the heterogeneous distribution of mineral phases underscores the influence of local metabolic activity on CaCO_3_ formation. Regions dominated by CaCl_2_/Ca ads EPS (spots a and d) likely represent areas of previously active fungal growth before inactivation, where EPS serves as a Ca^2+^ reservoir and nucleation template. In contrast, regions dominated by calcite suggest a progressive mineral transition, consistent with nonclassical crystallization pathways, where amorphous precursor phases transition toward a more thermodynamically stable polymorph.^42^

By capturing spatial heterogeneity in fungal systems and the molecular-level interaction between organic matrices and minerals at subcellular resolution, STXM/NEXAFS addresses key knowledge gaps in fungal biomineralization. However, some challenges remain, including spectral noise in very low-density regions and sample thickness effects. Additionally, the potential loss of fragile fungal cell surface associated with minerals during washing and fixation may have, to some extent, influenced the C chemistry data or led to an underestimation of the EPS role in CaCO_3_ precipitation. These highlight areas for methodological refinement.

These findings suggest that fungal biomineralization may occur via both microbially induced calcium carbonate precipitation (MICCP), where fungal metabolic activity induces CaCO_3_ precipitation, and microbially influenced mineralization (MIM), where organic matrices like EPS serve as nucleation sites. The coexistence of these pathways highlights the versatility of fungi in CaCO_3_ precipitation.

This study bridges knowledge gaps across scientific disciplines and contributes to the development of sustainable, resilient materials, while aligning with global efforts to reduce CO_2_ emissions. The dominance of the calcite polymorph in fungal-induced CaCO_3_ biominerals validates the feasibility of applying fungi in concrete self-healing, as stable calcite precipitation offers long-term durability and stability against structural degradation. However, the observed heterogeneity, including less stable CaCO_3_ and non-CO_3_–Ca phases, highlights the need to optimize fungal growth conditions and application methods for field-scale implementation. Further validation in real concrete is crucial to assess fungal performance under diverse structural and environmental conditions.

## Conclusions

This study demonstrates the capability of synchrotron-based STXM/NEXAFS in elucidating nanoscale mechanisms in fungal biomineralization. Detailed Ca L-edge and C K-edge analyses identified calcite as the dominant biomineral phase, precipitated across analyzed samples from three urease-positive fungal strains, with a minor contribution from aragonite detected. Certain calcite-dominated regions contained considerable amounts of non-CO_3_–Ca species, identified as residual CaCl_2_ particles or Ca adsorbed onto EPS. These findings also reveal nanoscale heterogeneity in biomineralization patterns, especially for fungal species 2, likely influenced by fungal metabolism-induced changes in pH and the carbonates system, local availability of Ca^2+^ ions in certain nanoregions, and the presence of EPS interactions. By spatially resolving polymorph distributions at subcellular scales, this study bridges critical knowledge gaps. It advances the understanding of fungal roles in CaCO_3_ biomineralization. Moreover, the observed preferential precipitation of thermodynamically stable calcite highlights the potential of fungal-induced biomineralization for self-healing concrete applications.

## Supplementary Material



## Notes

Composition of the fungal strain screening process, reference NEXAFS spectra for carbon (K-edge) and calcium (L-edge), additional SEM and STXM image, and NEXAFS data processing (PDF)
