# Supplementary material for: Nanoscale Characterization of Fungal-Induced CaCO3 Precipitation: Implications for Self-Healing Concrete
Source: ACS Appl Mater Interfaces. 2025 Jun 20;17(26):37648–56. doi: 10.1021/acsami.5c07137 (PMC12232265; doi:10.1021/acsami.5c07137)
Supplement: Supplementary file 1 [file am5c07137_si_001.pdf]

Supporting Information

Nanoscale Characterization of Fungal-Induced $CaCO_3$ Precipitation: Implications for Self-Healing Concrete

J.R. Marius Tuyishime,[*,†,‡] Edith C. Hammer,[‡] Martí Pla-Ferriol,[‡] Karina Thånell,[†] Carl Alwmark,[‡] Sophie van Velzen,[‡] Dimitrios Floudas,[‡] Rasa Platakyte,[‡] Martin Obst[§] Hanbang Zou[*,‡]

[†]MAX IV laboratory, 224 84 Lund, Sweden

[‡]Lund University, 223 62 Lund, Sweden

[§]BayCEER, University of Bayreuth, 95448 Bayreuth, Germany

*Email: jr_marius.tuyishime@maxiv.lu.se.

*Email: hanbang.zou@biol.lu.se.

Fungal strain isolation from environmental samples

Environmental samples were obtained from an abandoned limestone quarry in Skåne, Sweden, by swabbing different soils with sterile swabs. The samples were placed in tubes containing 10 mL of sterile water. The tubes were then brought to the laboratory of the Microbial Ecology research group, Department of Biology, Lund University. Upon arrival, the tubes were vortexed for 10 minutes. A subsample of 0.1 ml was transferred to a new tube, diluted 100 times by adding 9.9 ml of sterile water, and stored at 4 ºC. The next day, 0.1 ml of each sample was added to a petri dish containing Christensen's Urea Agar (CUA) containing Tetracycline (100 mg/l), to isolate urease-positive fungal strains. Those plates were incubated at 20ºC for 15 days, and fungal colonies presenting urease activity were then recultured into new CUA plates to select those with the highest enzymatic activity. Finally, the selected strains were transferred to YMG (Yeast-Malt-Glucose) agar plates for maintenance.

Fungal-induced calcium carbonate precipitation test

From axenic cultures of each fungal candidate, a 5 mm diameter agar plug was extracted and transferred to a 100 mL liquid culture medium (Christensen's Urea Broth) containing Ca^{2+} ions, introduced via the addition of 50 mM $CaCl_2.4H_2O$ solution. All liquid cultures were prepared inside 250 mL sterile flasks, sealed with aluminum foil, and incubated at 20 ºC with agitation for 14 days. Finally, a small portion of the floating mycelium was extracted for further processing.

Figures

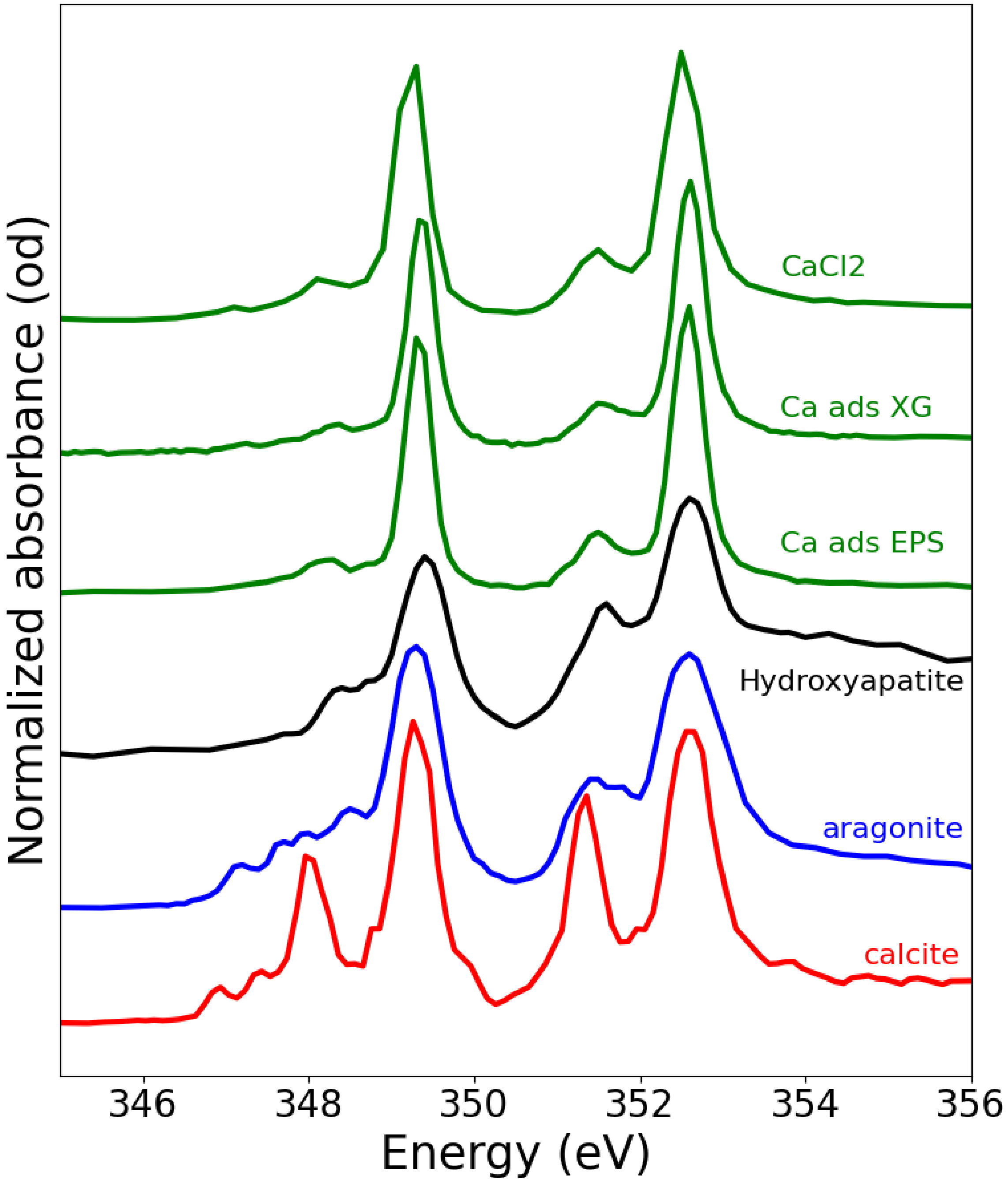

Figure S1: Ca L-edge NEXAFS for relevant Ca references normalized to an absolute linear absorbance scale (optical density per nm effective thickness) (Hanhan, Smith et al. 2009, Obst, Dynes et al. 2009), except for $CaCl_2$, which was obtained as internal standard from image stacks.

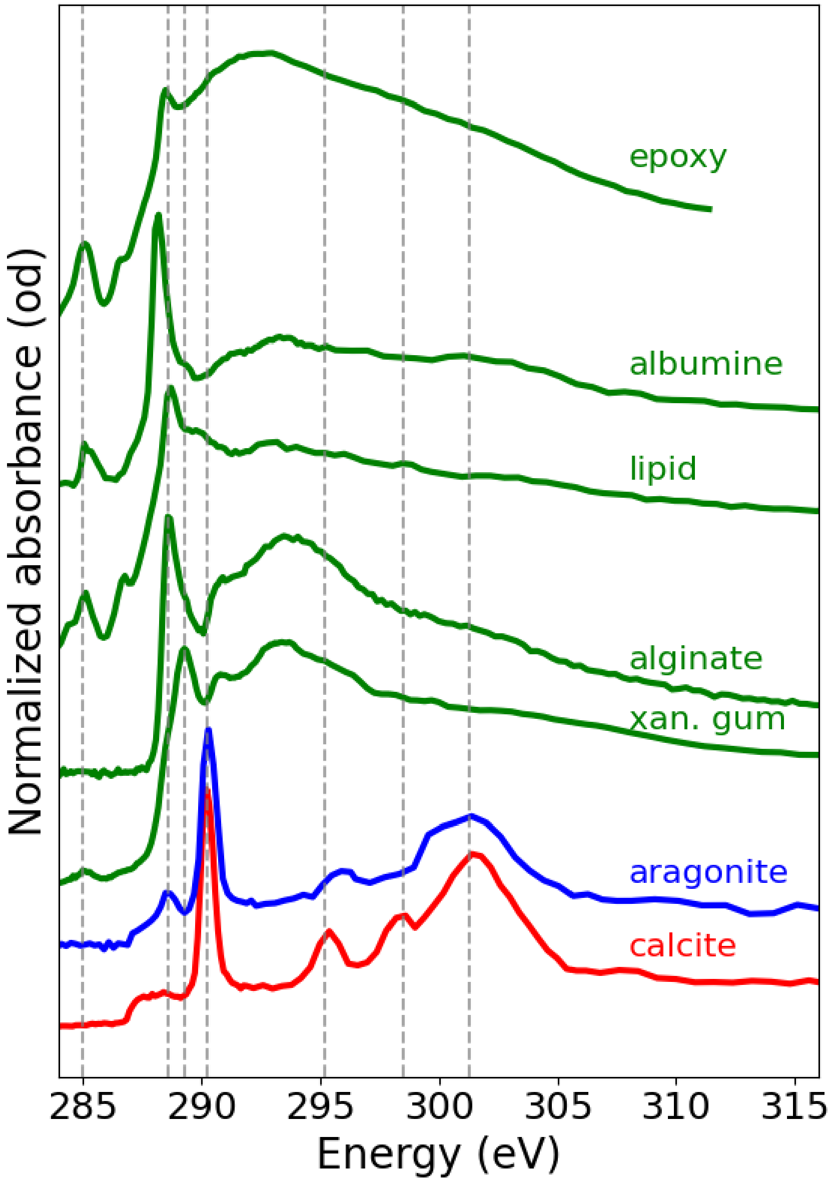

Figure S2: C K-edge NEXAFS for relevant C references normalized to an absolute linear absorbance scale (optical density per nm effective thickness) (Hanhan, Smith et al. 2009, Obst, Dynes et al. 2009).

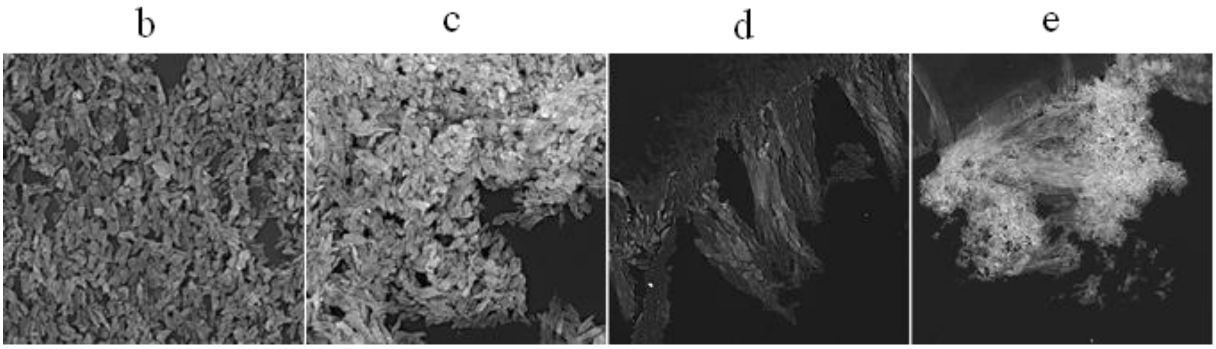

Figure S3: Individual SEM images showing morphological features of a thin section of $CaCO_3$ biominerals produced by fungal species 2. (b), (c), (d), and (e) indicate areas in the thin section where STXM data were collected to analyze Ca at the L-edge and C at the K-edge. Image of spot (a) is missing.

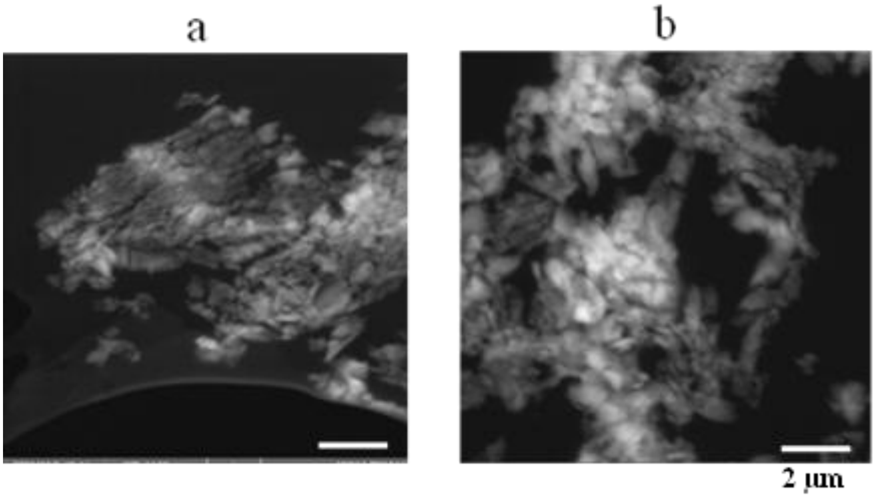

Figure S4: Individual SEM images showing morphological features of a thin section of $CaCO_3$ biominerals produced by fungal species 3. Pannels (a), (b) indicate spots in the thin section where STXM data were collected to analyze Ca at the L-edge and C at the K-edge.

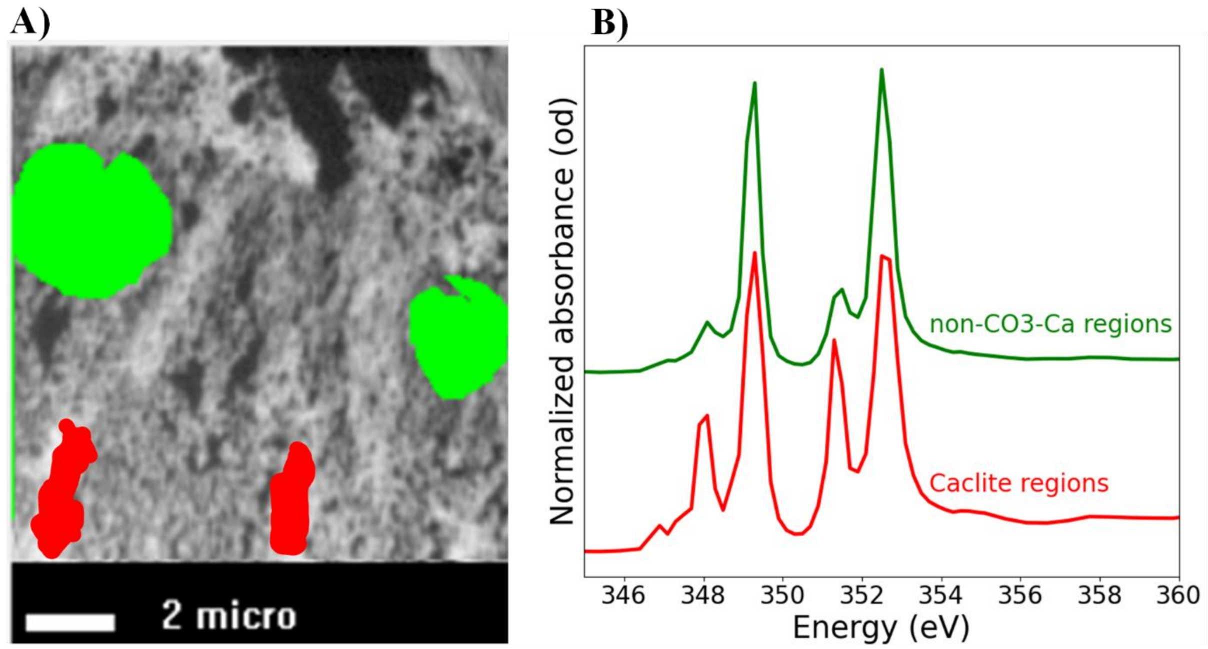

Figure S5: **A)** Average STXM image stack over the Ca L-edge (340–360 eV) collected from fungal species 2, spot (a). Color-coded regions highlight areas dominated by calcite (red) and non-CO_3-Ca species (green). **B)** Average Ca L-edge NEXAFS spectra extracted from selected pixels in regions dominated by calcite (red) and non-CO_3-Ca species (green) in the same spot. The stack fit showed quantitative chemical composition and spatial distribution of these Ca species across the entire area, corresponding to spot (a) in Figure 5A of the main manuscript.

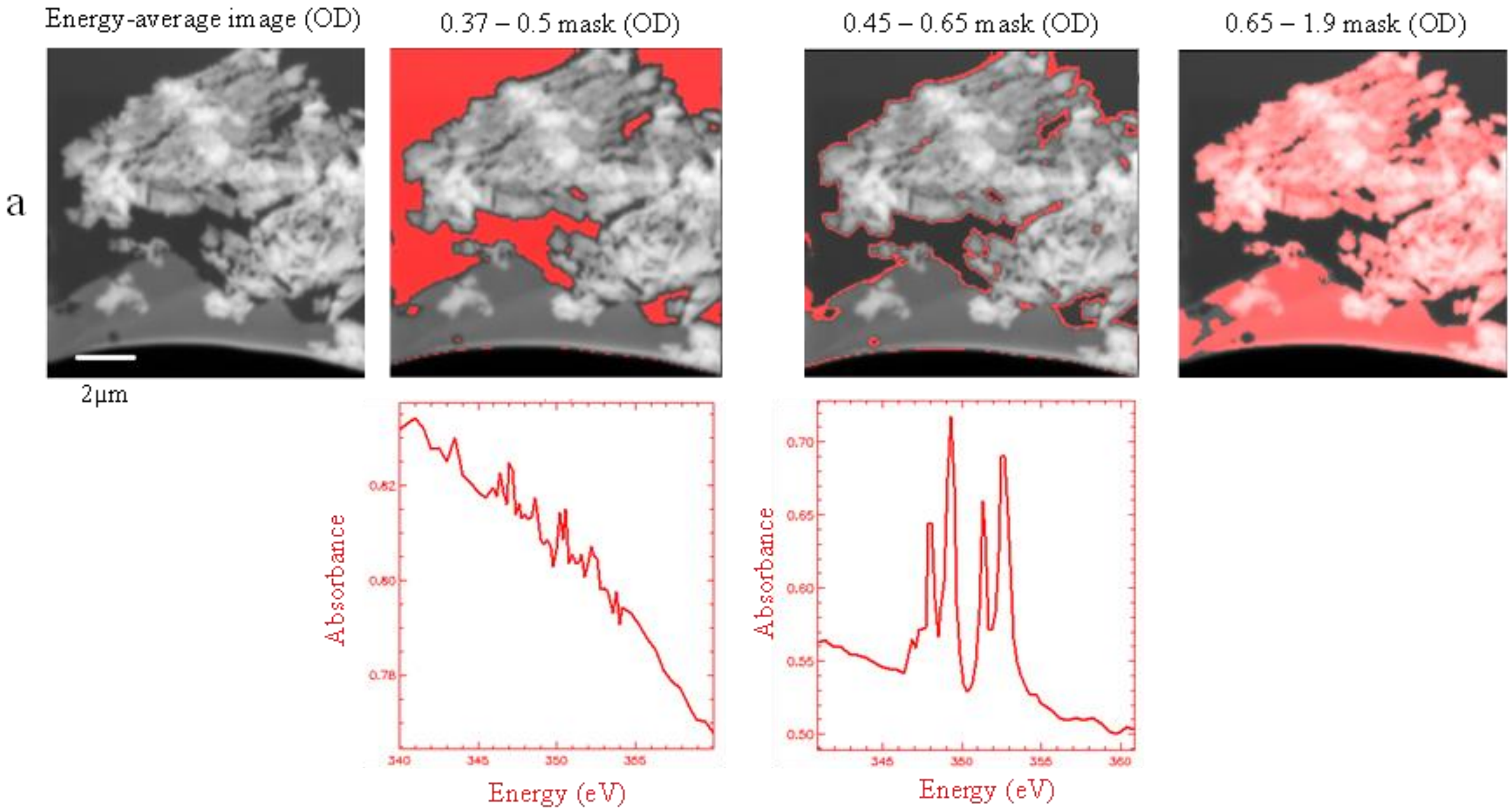

Figure S6: STXM image masks processing and spectral analysis. (*top row*): averaged STXM image (in optical density (OD)) taken over Ca L edge (340 – 360 eV) in spot (**a**) from $CaCO_3$ biominerals produced by fungal species 3, followed by thickness (in terms of OD)- based masked regions (red) (0.37 – 0.50, 0.5 – 65, and 0.65 – 1.9). (*Bottom row*): Corresponding NEXAFS spectra extracted from first two masked regions, highlighting variations in absorbance. The region with 0.37 – 0.50 OD showed a very high noise level with no detectable Ca signal, while 0.5 – 65 OD region marked the onset of Ca L-edge signal. The spectra from thicker regions (0.65 – 1.9 OD) were not included.

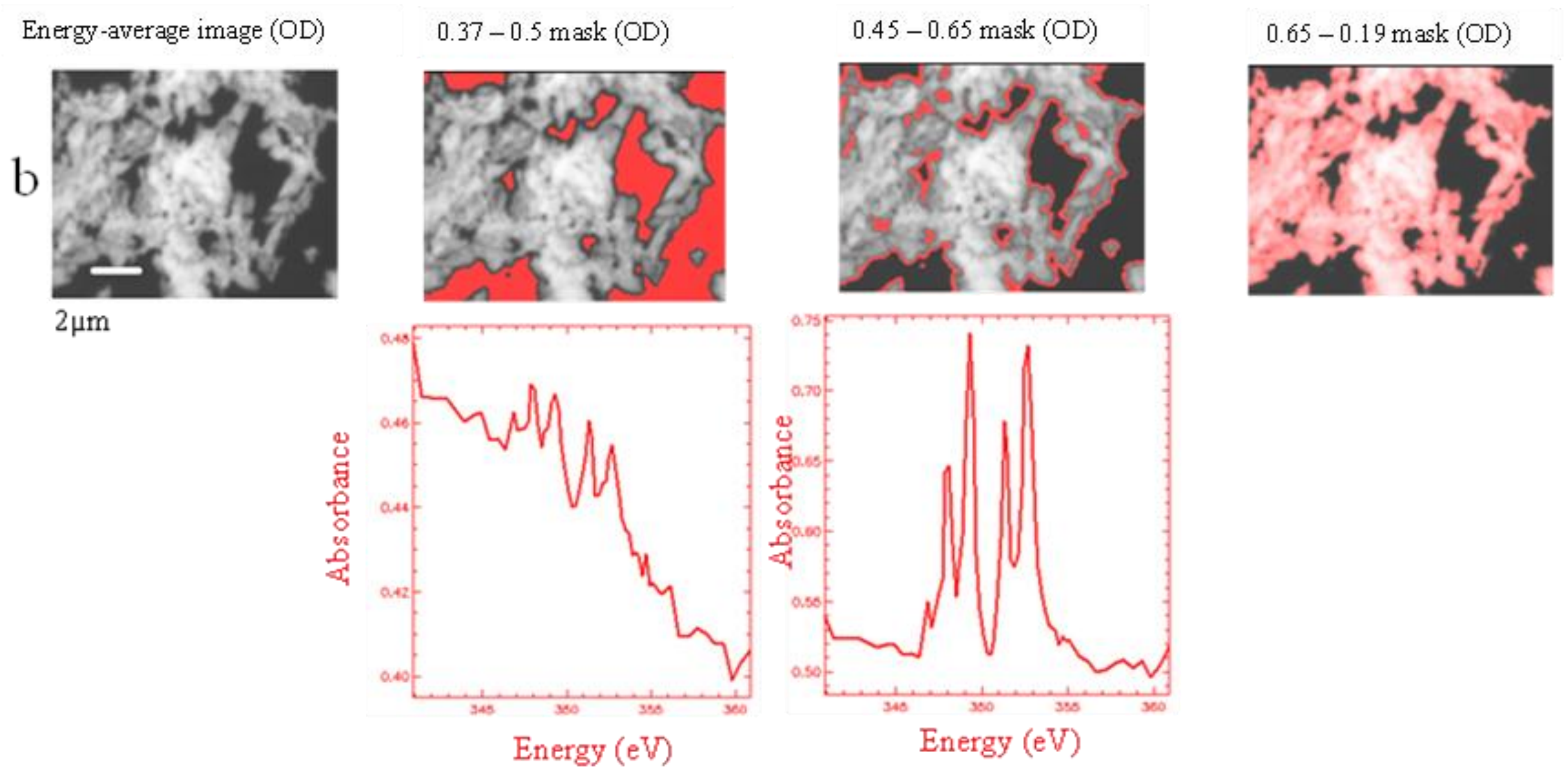

Figure S7: STXM image masks processing and spectral analysis. (top row): averaged STXM image (in optical density (OD)) taken over Ca L edge (340 – 360 eV) in spot (**b**) from $CaCO_3$ biominerals produced by fungal species 3, followed by thickness (in terms of OD)-based masked regions (red) (0.37 – 0.50, 0.5 – 65, and 0.65 – 1.9). (Bottom row): Corresponding NEXAFS spectra extracted from first two masked regions, highlighting variations in absorbance. The region with 0.37 – 0.50 OD showed a very high noise level with no detectable Ca signal, while 0.5 – 65 OD region marked the onset of Ca L-edge signal. The spectra from thicker regions (0.65 – 1.9 OD) were not included.

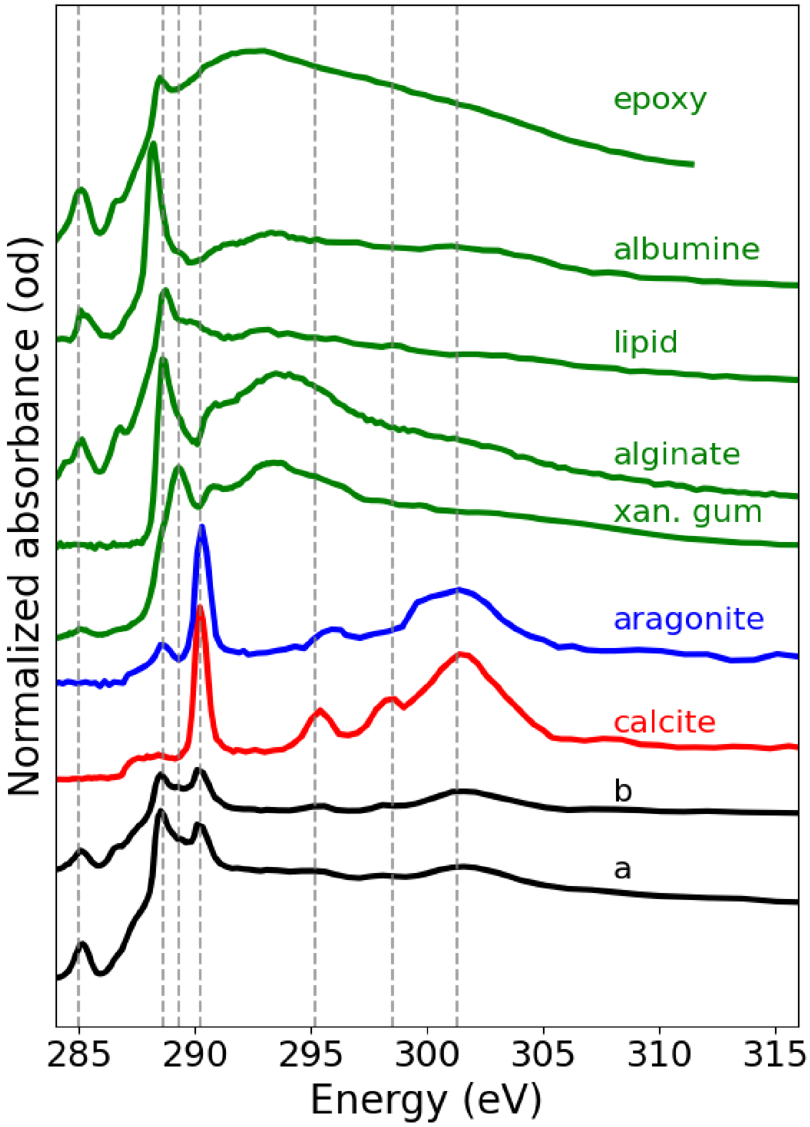

Figure S8: C K-edge NEXAFS data for $CaCO_3$ minerals produced by fungal species 3: (a) and (b) show average spectra (black) extracted from non-absorption saturation regions of two analyzed spots. The sample data are compared with reference C spectra normalized to an absolute linear absorbance scale (OD per nm effective thickness) (Dynes, Lawrence et al. 2006, Hitchcock, Dynes et al. 2009, Miot, Benzerara et al. 2009), except for epoxy resin, which was obtained as internal standards from image stacks.
